# Translational Potential of Immune Tolerance Induction by AAV Liver-Directed Factor VIII Gene Therapy for Hemophilia A

**DOI:** 10.3389/fimmu.2020.00618

**Published:** 2020-04-28

**Authors:** Benjamin J. Samelson-Jones, Valder R. Arruda

**Affiliations:** ^1^The Children’s Hospital of Philadelphia, Philadelphia, PA, United States; ^2^Perelman School of Medicine, University of Pennsylvania, Philadelphia, PA, United States; ^3^Raymond G. Perelman Center for Cellular and Molecular Therapeutics, Philadelphia, PA, United States

**Keywords:** hemophilia A, inhibitors, adeno-associated virus, gene therapy, anti-drug antibodies, immune tolerance

## Abstract

Hemophilia A (HA) is an X-linked bleeding disorder due to deficiencies in coagulation factor VIII (FVIII). The major complication of current protein-based therapies is the development of neutralizing anti-FVIII antibodies, termed inhibitors, that block the hemostatic effect of therapeutic FVIII. Inhibitors develop in about 20–30% of people with severe HA, but the risk is dependent on the interaction between environmental and genetic factors, including the underlying *F8* gene mutation. Recently, multiple clinical trials evaluating adeno-associated viral (AAV) vector liver-directed gene therapy for HA have reported promising results of therapeutically relevant to curative FVIII levels. The inclusion criteria for most trials prevented enrollment of subjects with a history of inhibitors. However, preclinical data from small and large animal models of HA with inhibitors suggests that liver-directed gene therapy can in fact eradicate pre-existing anti-FVIII antibodies, induce immune tolerance, and provide long-term therapeutic FVIII expression to prevent bleeding. Herein, we review the accumulating evidence that continuous uninterrupted expression of FVIII and other transgenes after liver-directed AAV gene therapy can bias the immune system toward immune tolerance induction, discuss the current understanding of the immunological mechanisms of this process, and outline questions that will need to be addressed to translate this strategy to clinical trials.

## Introduction

Hemophilia A (HA) is an X-linked bleeding disorder due to inherited deficiency in coagulation factor VIII (FVIII) activity (1, 2). Until recently, treatment in the developed world involved the intravenous administration of FVIII concentrates to treat or prevent bleeding. Recently, a FVIII-mimetic that is delivered subcutaneously, emicizumab, has been approved as prophylactic treatment for HA to prevent bleeding (3–6). Emicizumab is the first approved non-factor therapy for HA, but several others are in clinical development (7, 8). Other novel treatments for HA in clinical studies include several gene therapy approaches (9–11).

The major complication of treatment with FVIII concentrates is the development of neutralizing anti-FVIII antibodies, termed inhibitors, which substantially increase the mortality and morbidity of HA (12–19). Inhibitors are clinically quantified in Bethesda Units (BU), where 1 BU neutralizes 50% of normal FVIII activity. High-titer inhibitors greater than 5 BU generally prevent administered FVIII from having a therapeutic effect (20). On a research basis, anti-FVIII antibodies can also be quantified by immunosorbent assays that measure both non-neutralizing and neutralizing antibodies. Clinically, decreased FVIII recovery (expected peak level after FVIII protein administration) and half-life are also used as markers for non-neutralizing antibodies and evidence of an anti-FVIII immune response even in the absence of measurable Bethesda titers. Bypassing agents, which circumvent the inhibitor to provide hemostasis, are required to treat or prevent bleeding in high-titer inhibitor patients (21, 22). Due to their mechanism of action, emicizumab and other non-factor therapies are also effective in the presence of high-titer inhibitors, though they are currently only studied to prevent bleeding (5).

Inhibitors develop in about 20–30% of patients with severe hemophilia A (<1% normal FVIII activity) and in about 10% in patients with non-severe hemophilia A (1–40% normal FVIII activity) (23, 24). The risk of inhibitor development for the former is highest during their initial FVIII exposure days (25), while it is relatively constant for the latter (23). Both genetic and environmental factors influence the risk of inhibitor development (25–29). A major genetic determinant of inhibitor risk is the underlying hemophilia-causing FVIII-gene (*F8*) mutation (28). Patients with *F8* mutations resulting in the expression of some FVIII cross-reactive material (CRM), such as missense or small in-frame deletions or insertions, are less likely to develop inhibitors while CRM-negative patients with large *F8* deletions are more likely to develop inhibitors (28). Environmental factors include the manufacturing process and type of factor product, timing of first factor exposure, factor dosage, and clinical situations that result in immunological “danger signals” (24–26, 29).

Treatment of HA patients with inhibitors includes the prevention and treatment of bleeds (20, 30) and, historically, eradication of the inhibitor via the immune tolerance induction (ITI) regimens (30–33). ITI is the frequent regular infusion of FVIII concentrates over extended period-of-time (often years) with a goal of sufficiently decreasing the inhibitor to allow for the use of therapeutic FVIII, as nothing provides as effective hemostasis as FVIII in the absence of an inhibitor (20). The frequency and the dose of FVIII in ITI remain debatable, but the dosing regimens of daily or every-other-day from the International ITI Study (33) are often used (20, 30). However, ITI is only successful in about 60% of patients (32, 33). The underlying mechanism is likely to be peripheral immune tolerance induction where the activity of anti-FVIII immune cells is suppressed through tolerogenic interactions in the periphery, rather than central immune tolerance where the anti-FVIII immune cells are eliminated prior to leaving either the thymus or bone marrow.

The recent advent of emicizumab, which provides significantly improved bleeding prophylaxis compared to other bypassing agents (5, 6), has raised the question of whether inhibitor eradication remains necessary in the management of inhibitor patients (34, 35). Though the clinical consensus to this question is still forming, many experts continue to recommend ITI for new inhibitors (36) given the ongoing concerns about thrombotic complications in inhibitor patients on emicizumab receiving high cumulative doses of the bypassing agent activated Prothrombin Complex Concentrates for break-through bleeding (5, 37, 38). Long-term follow up is needed to define the “real world” safety and efficacy of indefinite emicizumab compared standard ITI.

The limited success rate of current ITI approaches has driven the pre-clinical investigations of several novel ITI strategies (39), including gene therapy approaches (40). Multiple adeno-associated viral (AAV) vector gene therapies for HA without inhibitors are in clinical development, as summarized in Table 1 (9–11). These drugs all direct the therapeutic FVIII-gene to hepatocytes expression. Though the goal of these studies is to achieve durable therapeutically relevant FVIII levels, emerging preclinical data suggest the liver-directed gene therapy can utilize the liver tolerance effect (41) to induce immune tolerance to the transgene-product (40, 42, 43). Here we review the preclinical data supporting the hypothesis that AAV liver-directed gene therapy can induce immune tolerance to FVIII and present the open questions that need to be considered when translating this approach to clinical trials.

**TABLE 1:** Current FVIII AAV liver-directed gene therapy products for HA in clinical development.

**Name**	**Sponsor**	**Vector serotype**	**Transgene^‡^**	**Manu-facturing**	**Phase**	**FVIII range (% normal)**	**ClinicalTrials.gov Identifier**
Valoctocogene	Biomarin	AAV5	FVIII-SQ	Baculovirus/Insect cells	3	19–164	NCT03370913
roxaparvovec (BMN-270)							NCT03392974
SPK-8011	Spark	LK03	FVIII-SQ	Plasmid/Mammalian cells	1/2	<5–49	NCT03003533
SPK-8016	Spark	*NA*	FVIII-SQ	Plasmid/Mammalian cells	1/2	*NA*	NCT03734588
AAV2/8-HLP-FVIII-V3	UCL	AAV8	FVIII-V3	Plasmid/Mammalian cells	1/2	6–69	NCT03001830
SB-525	Sangamo	AAV6	FVIII-SQ	Baculovirus/Insect cells	1/2	4–150	NCT03061201
SHP654 (BAX888)	Shire	AAV8	FVIII-SQ	Plasmid/Mammalian cells	1/2	*NA*	NCT03370172
BAY 2599023 (DTX201)	Bayer	AAVhu37	FVIII-SQ	Plasmid/Mammalian cells	1/2	5–17	NCT03588299

*^‡^See (69) and reference therein for details about FVIII variants used in gene therapy. NA, not available; UCL, University College of London.*

## The Liver Tolerance Effect in Liver-Directed Gene Therapy

### The Liver Tolerance Effect

The liver is a tolerogenic organ (41, 42, 44–46). Its specialized immune system limits immune reactivity against the constant flux of digested food-products as well as antigens from commensal microorganisms, while simultaneously safeguarding against gastrointestinal pathogens. From a therapeutic perspective, the liver tolerance effect was first recognized in studies of an outbred porcine liver transplant model where some allografts achieved long-term survival without immunosuppression (47); similar results were subsequently reported in other *in vivo* transplant models (48, 49). Moreover in animal studies, liver allotransplants also promote the immunological tolerance to other organ allografts from the same donor (47), and tolerance to renal and small bowel transplants is enhanced if the venous blood drainage of the grafts is through the portal system (50). Clinically, immunosuppression can be safely withdrawn in about 20% of liver-transplant patients (51), which is not achievable in other solid organ transplant patients.

The liver tolerance effect is also exploited by hepatotropic pathogens (44–46, 52). The *Plasmodium* species responsible for malaria initially target the liver after being delivered by an infected mosquito and then mature and replicate sheltered within hepatocytes before being released back into the blood stream. Malaria remains one of the most deadly human pathogens responsible for millions of annual deaths and has resisted effective vaccination strategies to date. Likewise, the protolerogenic environment of the liver likely impedes an effective adaptive anti-viral response in chronic infections by hepatitis B and hepatitis C virus (HCV) (52). Viral hepatitis remains a major source of morbidity and mortality especially in the developing world and in people with hemophilia (53, 54).

### The Liver Tolerance Effect in Liver-Directed Gene Therapy for Hemophilia B

The liver tolerance effect can also be exploited therapeutically by liver-directed gene therapy to induce immune tolerance to the transgene product (40, 42, 43). This was first demonstrated in HB mice that were tolerized to human (h) factor IX (FIX) after AAV gene therapy (55), but has subsequently been demonstrated in several other disease models including HA, which is discussed in detail below. In naïve HB mice, administration of liver-directed AAV (55) or lentiviral (LV) (56) vectors encoding hFIX induces immune tolerance that is resistant to subsequent hFIX immunizations. Interestingly, induction of immune tolerance with LV vectors requires the use of genome regulatory elements that prevented transgene expression in immune cells (56), suggesting that the tolerogenic bias of liver-directed gene therapy can be overwhelmed by non-liver transgene expression. In addition to induction of immune tolerance in naïve HB mice, both liver-directed AAV and LV hFIX gene therapy can also eradicate preexisting anti-hFIX antibodies and induce immune tolerance in hFIX-immunized HB mice (57, 58).

Similar outcomes have also been observed in canine models of severe HB. Colonies of naturally occurring HB dogs with distinct *F9* mutations provide highly informative models for evaluating immune responses to therapeutic canine (c) FIX. HB dogs with a *F9* null mutation are inhibitor-prone and typically develop an inhibitor after a single administration of cFIX concentrate (59), while HB dogs with a *F9* missense mutation are non-inhibitor-prone and very rarely develop inhibitors against cFIX, usually only in the context of a pro-inflammatory stimulus (60, 61). The use of cFIX protein, canine plasma, and cFIX encoding vectors allows the immune response to be interrogated in a species-specific manner.

No evidence of an anti-cFIX immune response has been reported after AAV (59, 62–65) or LV (66) liver-directed gene transfer with cFIX in 11 and 3 non-inhibitor-prone HB dogs, respectively, though only 1 dog has been subsequently challenged with canine plasma for bleeding (64). Moreover, AAV liver-directed gene therapy with cFIX successfully tolerized 5 out 6 naïve inhibitor-prone dogs that typically form inhibitors after cFIX protein exposure (59, 67). In this study, the immune tolerance was demonstrated to be maintained despite subsequent cFIX protein exposures in all 4 dogs evaluated, while the 5th animal was not challenged (67, 68). The singe naïve HB dog that developed an inhibitor notably had a hemolytic anemia associated with liver iron-loading and consequentially liver-fibrosis, both of which were seen on liver histology at necropsy (59). The course of this single naïve inhibitor-prone dog that was not tolerized after AAV liver-directed gene therapy with cFIX may be the exception-that-proves-the-rule as his unrelated liver pathology likely disrupted the liver tolerance effect.

Liver-directed AAV gene therapy has also eradicated preexisting anti-cFIX antibodies and induced immune tolerance in an inhibitor-prone HB dog (Wiley) (67). In this study, an inhibitor-prone HB dog was previously exposed to hFIX and developed an anti-hFIX response with cross-reactive neutralization of cFIX activity. AAV liver-directed gene transfer with the hyperactive cFIX variant, Padua (69, 70), resulted in eradication of the anti-cFIX antibodies and disappearance of the anti-hFIX neutralization within 3 months of vector administration, though a minimal residual non-neutralizing anti-hFIX response remained detectable years after vector administration (67). The persistence of non-neutralizing anti-hFIX antibodies suggests that the immune tolerance induced after liver-directed AAV gene therapy is transgene specific. Nonetheless, this dog remained tolerant to cFIX even after exposure to cFIX-WT with over 3 years of ongoing observations (*VRA, unpublished data*).

### Immune Mechanisms of the Liver Tolerance Effect

**FIGURE 1:**
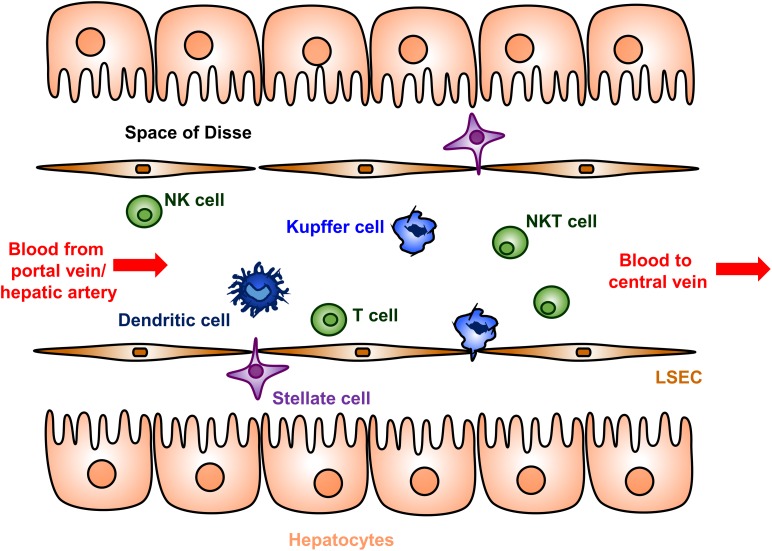
Cellular anatomy of the liver sinusoid. Blood enters the liver from the portal vein and hepatic artery, flows through a network of sinusoids schematically represented here, and then exits via the hepatic central vein. The sinusoids are lined by a fenestrated layer of specialized liver sinusoidal endothelial cells (LSECs), which are the endogenous site of most FVIII secretion. The LSECs shield the hepatocytes from direct sinusoidal blood flow by creating the Space of Disse, which contains the stellate cells. Dendritic cells, Kupffer cells, T cells, natural killer (NK) cells, and natural killer T (NKT) cells are abundantly present in the sinusoidal lumen. Hepatic antigen presenting cells include dendritic cells, Kupffer cells, LSECs, stellate cells, and hepatocytes. The hepatocyte microvilli can interact with luminal T cells.

Several complementary immunological mechanisms underlying the liver tolerance effect have been described [reviewed in detail in (41, 42, 44, 45, 71–73)]. The liver microenvironment contains unique populations of both antigen presenting cells and suppressor and effector T cells (Figure 1). Hepatic antigen presenting cells include conventional types such as dendritic cells, but also unique non-conventional antigen presenting cells that include liver sinusoidal endothelial cells (LSECs), Kupffer cells, stellate cells, and hepatocytes. Presentation of antigens by these specialized hepatic antigen presenting cells typically results in diminished effector T cell activity and/or increased suppressor activity of regulator T cells (Tregs), both of which promote immune tolerance.

Interactions between these hepatic antigen presenting cells and effector T cells often results in functional inhibition or cell death of the effector T cell. Low-expression of major histocompatibility complex (MHC) and co-stimulatory molecules on several types of hepatic antigen presenting cells contributes to abortive activation and result in T cell anergy in an antigen-specific manner (42, 73, 74). The absent production of the costimulatory cytokine IL-12 by Kupffer and dendritic cells also contributes to this process. Additionally, hepatic antigen presenting cells actively express a number of molecules that suppress effector T cell activity. Both Kupffer cells and hepatic dendritic cells produce indoleamine 2,3 dioxygenase (IDO) and prostaglandin E_2_, which inhibit T cell proliferation through distinct pathways (42, 74). Dendritic cells, LSECs and AAV transduced hepatocytes express Fas-L (CD95L), which allows for the direct deletion of effector T cells (42).

The liver microenvironment also promotes Treg activation and proliferation. Abundant Tregs are noted in liver allografts in mouse models and their depletion result in a loss of tolerance (75). Secretion of the suppressor cytokine IL-10 by LSECs, Kupffer cells, and hepatic dendritic cells promote Treg proliferation as well as conversion of effector T cells to Treg (42, 74). The decrease in the inhibitor titer after AAV gene therapy in the HB dog with a pre-existing inhibitor (Wiley) was associated the increasing IL-10 levels (67). Hepatocyte production of transforming growth factor-β similarly promotes Treg proliferation (76).

## Immune Tolerance Induction After Liver-Directed Gene Therapy in Preclinical Hemophilia a Models

### Immune Tolerance Induction in Naïve Hemophilia A Animal Models

Immunocompetent HA mice generally develop a xenoprotein immune response against hFVIII or cFVIII after exposures via protein administration or gene therapy (77, 78), and typically not against mouse (m) FVIII (79, 80). As such, preclinical efficacy studies of non-murine FVIII gene therapy have typically utilized immunosuppression or HA mice crossed with an immunodeficient model (81, 82).

However, AAV-liver directed gene therapy with hFVIII has been demonstrated to be able to induce immune tolerance that is resistant to subsequent protein challenges in HA mice (83). The ability of this approach to induce tolerance to hFVIII was dependent on both the HA strain background and the expressed FVIII level (83, 84). Immune tolerance to hFVIII in HA mice increased with higher hFVIII levels after AAV gene therapy, achieved either through codon optimization (83) or higher vector doses (84), though the controls were likely at sub-therapeutic hFVIII levels (<1% normal).

In contrast to the murine model, outbred severe HA dogs predictably develop inhibitors against cFVIII (85–88). These dogs have a *cF8* mutation analogous to the human *F8* intron-22 inversion, the most common causative severe HA mutation in patients (89, 90). Intriguingly, the risk of inhibitor development in these HA dogs is in part inherited through yet undefined non-*cF8* genes as the propensity of inhibitor development is increased in the progeny of certain outside breeders (86, 88). About 30% of these inhibitor-prone HA dogs, either at the colony at Queens University (QU) or University of North Carolina at Chapel Hill (UNC-CH) colony, develop anti-cFVIII inhibitors (86, 91).

None of the 15 non-inhibitor-prone HA dogs that have received cFVIII AAV-liver-directed gene therapy without immunosuppression have made an anti-cFVIII immune response (92–94). At least 6 of these animals were subsequently challenged with recombinant cFVIII protein (25 U/kg weekly for 4 weeks) 1–5 years after vector administration and did not display an anti-FVIII immune response. Recovery of the administered cFVIII protein was similar to that of naïve dogs, which confirmed complete tolerance to cFVIII (94).

However, 2 out of 9 inhibitor-prone HA dogs developed an inhibitor after cFVIII AAV-liver-directed gene therapy (91, 94, 95). A QU-housed HA dog Junior developed an inhibitor 2 weeks after administration of an AAV2 vector via the portal-vein with a peak titer of 9 BU at 4 weeks that became undetectable by week 9 (91). Similarly, an UNC-CH inhibitor-prone HA dog L51 developed an inhibitor within a week of intravenous administration of an AAV8 vector that peaked at 2.5 BU and became undetectable by week 7 (94). Notably, the eradication of L51’s inhibitor was stringently demonstrated to be immune tolerance by the lack of recurrent anti-cFVIII antibodies and expected recovery of challenges of recombinant cFVIII protein (25 U/kg weekly for 4 weeks) (94). Though limited by the small numbers, the experience of Junior and L51 suggest that AAV-liver-directed gene therapy can induce stringent immune tolerance to transgene-FVIII in large outbred models of HA.

However, the liver tolerance effect after liver-directed gene therapy in HA dogs can be disrupted in the setting of hepatotoxicity (96), similar to what was discussed above for HB dog models (59). In an early study using an adenoviral vector (rather than AAV), all 4 QU HA dogs that received liver-directed cFVIII gene therapy developed inhibitors in the setting of an early (0–4 weeks), presumably adenovirus-induced, hepatotoxicity manifested by over a 20-fold increase in liver enzymes (96). Both the increase in liver enzymes and the inhibitor titer appeared to be vector dose dependent with the 2 dogs that received the lower vector dose only developing low titer inhibitors (peak < 2 BU) that disappeared within 4 weeks; there was also no recurrence of the inhibitor after administration of canine FVIII cryoprecipitate (20 U/kg) in these 2 low-dose dogs (96). Though this study was conducted with a vector that is no longer translationally relevant for HA, the observations about the immune response in setting of acute hepatotoxicity may be germane for other gene therapy approaches.

Consistent with this hypothesis that hepatotoxicity can interfere with the pro-tolerogenic effects of liver gene therapy is the subsequent observation that none of the 3 the QU HA dogs that received liver-directed cFVIII gene therapy with a less toxic helper-dependent adenovirus vector concomitantly with steroid immunosuppression developed an anti-cFVIII immune response (97). A fourth dog in this study received cFVIII gene therapy under-the-control of a ubiquitous CMV promoter and did develop a high-titer inhibitor (peak 150 BU 1 week after vector administration) (97). This observation suggests that non-liver expressed cFVIII can potentially interfere with immune tolerance from liver-expressed transgene and is consistent with the observations from HB (56) and Pompe (98, 99) disease models.

The cumulative data from both small and large animal HA models indicates that liver-directed gene therapy, especially with AAV vectors that have minimal hepatoxicity in pre-clinical models, can induce immune tolerance in naïve animals to FVIII. Moreover, if anti-FVIII antibodies develop, the continued transgene expressed FVIII can eradicate the inhibitors and, in a limited number of examples, induce immune tolerance that is resistant to subsequent protein challenges.

### Inhibitor Eradication and Immune Tolerance Induction in Hemophilia A Inhibitor Models With Pre-existing Anti-FVIII Immune Response

The likely more challenging and clinically relevant scenario is the eradication of a pre-existing anti-FVIII immune response. We have previously reported on the successful immune tolerance induction in 4 inhibitor-prone HA dogs with pre-existing anti-cFVIII antibodies after AAV liver-directed gene therapy (Table 2) (100). In this study, 3 HA dogs from UNC-CH with peak historical inhibitor titers from 4 to 12 BU received cFVIII AAV8 liver-directed gene therapy. Notably, the anti-FVIII IgG and inhibiter titers in all 3 dogs disappeared by 5 weeks after vector administration (100).

**TABLE 2:** Summary of inhibitor eradication in hemophilia A dogs following cFVIII AAV liver-directed gene therapy.

			**Pre-gene therapy**		**Titer at vector (BU)**	**Post-gene therapy**		
**Dog**	**Age (year)**	**Weight (kg)**	**Inhibitor duration (wks)**	**Peak titer (BU)**		**Peak titer (BU)**	**Time to eradication (wks)**	**cFVIII activity (%)^‡^**
K01	1.7	20.1	32	12	3	7	5	1.5
K03	1	19.3	28	12	3	3	4	8.0
L44	0.7	16.0	28	4.5	2.2	2.2	4	1.5
Wembley	4.9	16.5	96	3.6	3.5	216	80	>1.5^†^

*Data from (100). ^†^VRA, unpublished observation. ^‡^Plateau level after inhibitor eradication.*

The fourth inhibitor-prone HA dog (Wembley from QU) with pre-existing anti-cFVIII antibodies treated similarly with cFVIII AAV8. At the time of vector administration, his cFVIII-inhibitor was 4 BU, which was similar to his peak titer. After vector administration, Wembley also had a large anamnestic response with dramatic increases in his anti-cFVIII IgG and inhibiter titer that peaked at 216 BU. However, both these parameters decreased until they became undetectable at about 80 weeks after vector administration (*VRA, unpublished observation*).

The post-gene therapy cFVIII activity after inhibitor eradications was between 1.5 and 8% of normal in these 4 dogs (100) (*and VRA, unpublished observation*). The immune tolerance of all 4 of these dogs was maintained despite multiple challenges with recombinant cFVIII protein that displayed typical pharmacokinetics in an ongoing study (100) (*and VRA, unpublished observation)*. Though Wembley was tolerized to cFVIII after cFVIII AAV liver-directed gene therapy, he never fully tolerized to hFVIII, his triggering antigen (*VRA, unpublished observation*). Similar to the above discussion for the HB dog models (67), Wembley’s course suggests that the immune tolerance of AAV liver-directed gene therapy is transgene specific. To date, cFVIII inhibitors have been eradicated in 6 HA dogs (2 dogs naïve discussed in section “Immune Tolerance Induction in Naïve Hemophilia A Animal Models” and 4 dogs with pre-existing inhibitors discussed in section “Inhibitor Eradication and Immune Tolerance Induction in Hemophilia A Inhibitor Models With Pre-existing Anti-FVIII Immune Response”) by cFVIII AAV liver-directed gene therapy, which also provided stringent immune tolerance and long-term therapeutically relevant cFVIII levels. This limited data suggests that AAV liver-directed gene therapy has translational potential for people with HA and inhibitors.

## Translational and Clinical Considerations

### Overview of Current Clinical Development of Liver-Directed Gene Therapy for Hemophilia A

It is an exciting time for gene therapy for HA with multiple liver-directed AAV-based products in clinical trials reporting therapeutically relevant to curative levels of FVIII (Table 1) (9–11). These current products are the result of decades of experimentation developing the necessary technologies and protocols to achieve these end points (101–104). These AAV drugs differ in their vector serotype, FVIII transgene, and manufacturing process. A variety of naturally occurring and bioengineered vector serotypes are being tested (105). Because the full-length FVIII gene exceeds the ∼4.7 kb packaging capacity of AAV vectors, all current approaches rely on B-domain deleted FVIII variants, either the standard FVIII-SQ (106) as is used in B-domain deleted protein products (e.g., Xyntha and Pfizer) or an engineered B-domain deleted variant (FVIII-V3) that is associated with increased FVIII expression (107). All transgenes have been codon-optimized, though likely by distinct algorithms.

To date, all trials stringently limit subjects at risk for inhibitor development by including only subjects with no history of inhibitors and more than 150 FVIII exposures, as inhibitors rarely develop after 50 exposure days in severe HA (25). However, a current study (NCT03734588) enrolling subjects meeting these inclusion criteria is described as a dose-finding Part 1 of a planned two part clinical development strategy focused on inhibitor patients.

No major sustained safety concerns have been reported in these studies, though several immunological obstacles to the AAV vector capsid continue to limit efficacy and widespread enrollment (108, 109). The long-term durability also remains an open question with trials reporting therapeutically relevant FVIII levels out to 3 years after vector administration, albeit with declining levels (110). Nonetheless, the success of these studies raises the question whether these drugs or similar products could be harnessed to induce immune tolerance in HA inhibitor patients by exploiting the liver tolerance effects (41, 42, 44–46).

There are several obstacles that need to be address to define the role of AAV liver gene therapy for HA complicated by the presence of inhibitors to FVIII. The experience with preclinical HA inhibitor models, especially the canine data, support the potential translation of AAV liver-directed gene therapy for people with HA and inhibitors. However, therapeutic translation of this approach will require several yet unresolved issues to be considered and specific preclinical studies designed to address unanswered questions (Table 3). Preclinical studies using the immunocompetent HA dog models that naturally develop inhibitors when exposed to cFIII in a species-specific manner will likely be the most informative.

**TABLE 3:** Translational considerations of AAV liver gene therapy for immune tolerance induction.

	**Goals**	**Potential solutions**
Hepatotoxicity: transient increase in liver enzymes	Limit AAV-capsid mediated cellular response or AAV-associated transient transaminitis of unknown origin	Lowering the vector dose and/or immunosuppression
Immunosuppression regimens	To prevent or to control ongoing liver toxicity and/or loss of transgene expression	Transient oral steroid, mycophenolate mofetil, tracrolimus, rapamycin (alone or in combination)* Avoid intense immunosuppression at the time of AAV administration
Expression of the transgene outside the liver	Optimize transgene expression Avoid inadvertent non-hepatocyte tissue with increased risk of immune response	Use of promoter and regulatory elements highly active in hepatocyte
Optimized FVIII function by developing variants of the transgene	Lowering the therapeutic vector dose with increased biological activity without detrimental immunogenicity	Systematic screening for FVIII variants and testing in both *in vitro* and *in vivo* studies

*Partial list of immunosuppressive drugs evaluated in AAV liver gene therapy [reviewed in (136)].

### Hepatotoxicity and Immunosuppression

Foremost, is the potential detrimental role of hepatotoxicity in successful immune tolerance induction. As discussed above, preclinical studies suggest that the tolerogenic bias of liver-directed gene therapy can be disrupted in the setting of hepatotoxicity. The hepatotoxicity in these disparate canine studies was secondary to either the specific vector employed (96) or underlying liver disease in the dog model studied (59). There are several liver pathologies that occur in HA patients receiving AAV liver-directed gene therapy that may impact the liver tolerance effect.

Though rates of viral iatrogenic infections including HCV have thankfully plummeted with improved blood donor screening, highly effective virucidal procedures, and the use of recombinant FVIII products, about a third of young men with severe HA have a history of HCV infection, while the rate in older men exceeds 90% (53). Historically, about 20–30% of HCV infected patients eventually developed end-stage liver disease, though the recent approval of highly effective oral anti-viral regimens will likely radically reverse this trajectory (54). Asymptomatic liver damage from chronic HCV infection theoretically could impair the liver-tolerance effect after AAV liver-directed gene therapy. Current AAV liver-directed gene therapy clinical trials exclude patients with active HCV or clinical evidence of liver disease. Moreover, there were no appreciable differences in the clinical course of 3 out of 9 published HA subjects that received AAV5-FVIII (valoctocogene roxaparvovec, BMN 270) that had a history of resolved HCV infection (111); similarly, there has been no appreciable difference in the clinical course of HB subjects with a history of HCV receiving AAV FIX gene therapy (112–114). Other liver diseases that occur in the HA population such as non-alcoholic fatty liver disease pose the same theoretical risk of potentially disrupting the liver tolerance effect. Preclinical studies may help inform on this concern, but stringent liver disease exclusion criteria will probably be advisable in initial clinical studies with inhibitor patients.

To date, some subjects in all AAV liver-directed gene therapy trials for HA or HB have demonstrated hepatotoxicity after vector administration as evidenced by asymptomatic elevated liver enzymes [reviewed in (7, 104)]. At least 2 distinct mechanisms likely contribute to these observations. In products utilizing an AAV2, AAV8, or similar serotype vectors manufactured in mammalian cells, a well described anti-AAV capsid cellular immune response can target transduced hepatocytes leading to loss of transgene expression, which can often, though not always, be controlled with immunosuppression with steroids (108, 109, 112–116). In products utilizing AAV5 vectors manufactured in insect cells, elevated liver enzymes have also been reported, but do not appear to be associated with loss of transgene or evidence of cellular immunity (104, 111, 117); the etiology of this latter hepatotoxicity is still being investigated. Studies of both these hepatotoxicities have been hampered by lack of preclinical models that fully recapitulate the clinical observations (118–122). The potential adverse role either of these hepatotoxicities could have on inducing immune tolerance after AAV liver-directed gene therapy is unknown. However, as both hepatoxicities appear to be vector-dose dependent, avoiding them by using the lowest effective vector dose is likely sensible. The potential immunological consequence of lowering the transgene FVIII level is discussed below in section “FVIII Level and Variant Transgenes.”

The use of immunosuppression in AAV liver-directed gene therapy for HA and inhibitors will require specific preclinical studies. In HA dogs with pre-existing inhibitors, there is an early increase in CD25 ^+^ FOXP3 ^+^ CD4 ^+^ cells, assumed to be Treg cells, within the first few days after cFVIII AAV liver-directed gene therapy that is associated with the decline in anti-cFVIII antibodies that is not observed in HA dogs without inhibitors receiving similar therapy (40, 100). However, intense immunosuppression with the anti-CD25 antibody daclizumab around vector delivery increases the anti-hFIX immune response in non-human primates (NHP) receiving hFIX AAV liver-directed gene therapy and is associated with a decrease in Treg cells (123). The rationale of this study was that daclizimab could potentially decrease effector T cell activity to limit the anti-AAV capsid immune response; however, daclizumab also depleted Treg cells leading to an unanticipated increase immunity against the transgene hFIX.

These findings are not restricted to anti-CD25 antibodies. More recently we uncovered that rabbit antitymoglobulin (ATG) may also be detrimental if administered around vector delivery (*BSJ and VRA, unpublished data, manuscript in preparation*). Combined, this limited data does suggest that there is an early critical time-period around vector administration where Treg cell expansion may be important for immune tolerance induction. As such, immunosuppression around the time of AAV vector delivery needs to be thoughtfully considered. Reassuringly, steroids promote antigen-specific immune tolerance HA mice against concomitantly administered hFVIII protein, likely through Treg-dependent mechanisms (124). However, more intense immunosuppression therapy needs to be stringently tested in large animal models to avoid unanticipated increased immunogenicity. Ongoing mechanistic studies evaluating cFVIII AAV gene therapy in HA dogs with pre-existing inhibitors may better inform on this issue.

There is also a concern about the potential for liver toxicity due to ectopic expression of FVIII in hepatocytes after gene therapy due to specific features of FVIII secretion (125–128). However, though 2 studies in HA mice did find evidence of the unfolded protein response (UPR) after AAV liver-directed gene therapy, there was no correlation between FVIII inhibitor formation and UPR markers (126, 127). Ectopic expression of human FVIII in mouse megakaryocytes is also associated with megakaryocyte apoptosis (129). However, the theoretical concern of UPR-induced hepatotoxicity further supports the initial use of the lowest effective vector dose.

### Non-liver Transgene Expression

Preclinical data in HA (97) and other disease models (56, 98, 99) highlight that non-liver expression of the transgene can disrupt the pro-tolerogenic effects of liver expression. Most preclinical and clinical studies for AAV liver-directed gene therapy have utilized liver-specific promoters based on the seminal work by Dr. Kathy Ponder (130); the prototypical expression cassette contains sequences from the truncated apolipoprotein E (ApoE) hepatic control region (HCR) and the alpha-1-anti-trypsin (A1AT) promoter. Though this construct mostly limits non-liver transgene expression, we are not aware of studies that have rigorously quantified the non-liver transgene expression in a preclinical model. The non-liver expression of a particular AAV product is a function of both the transduction efficiency of the vector to other tissues and the promoter efficiency in these tissues. Recent work using a new, highly sensitive experimental system demonstrated much broader AAV transduction as well as low level and/or transient transgene expression than previously appreciated (131). This study highlights the importance of preclinical studies in immune competent animal models with preexisting inhibitors to FVIII to specifically evaluate the efficacy of immune tolerance induction of a particular vector before moving into clinical studies.

### FVIII Level and Variant Transgenes

Studies in both HA (83, 84) and HB (55) mice have concluded that increasing transgene levels after AAV liver-directed gene therapy promote immune tolerance. In non-severe HA patients, increasing FVIII levels are associated with decreased bleeding frequency (132). However, as discussed above, these potential benefits should be weighed against complications that also likely increase with increasing vector dose that potentially could interfere with the liver tolerance effect, including AAV-associated hepatotoxicity and UPR in transduced hepatocytes.

By definition, antigen expression is required for immune tolerance induction. In the randomized International-ITI trial, higher and more frequent FVIII dosing was associated with a more rapid immune tolerance induction, but the lower and less frequent dose demonstrated similar overall success rate (33). The sustained cFVIII levels of the 6 HA dogs described above that had inhibitor eradication and immune tolerance induction with cFVIII AAV liver gene therapy ranged from 0.5 to 8% of normal (91, 94, 100). This canine data suggest that transgene FVIII levels in the low-mild range is sufficient for immune tolerance induction.

The use of variant FVIII transgene also needs to be carefully considered. Studies in HA and HB dogs suggest that the immune tolerance after AAV liver-directed gene therapy is transgene specific; gene therapy with cFVIII or cFIX transgenes resulted in immune tolerance toward the canine orthologs, while the anti-hFVIII or anti-FIX response persisted (67, 100). However, in this HB dog study, the transgene was the single-amino acid substituted hyperactive variant cFVIII-Padua (R338L) (69, 70) and the animal was also fully tolerized to wild-type (WT) cFIX as evidenced by the lack of neutralizing or non-neutralizing anti-cFIX-WT antibodies despite protein challenges with cFIX-WT (67) (*and VRA, unpublished observation)*. This suggests that though the immune tolerance is transgene specific, there can be cross-tolerance between highly similar transgenes, though the degree of similarity required is undefined. The degree of similarity between variant FVIII transgenes (69) and available protein FVIII products, therefore, should be considered when designing gene therapy products for inhibitor patients.

The concern is that if the inhibitor patient tolerized only to a variant-FVIII transgene that was immunological distinct from available FVIII protein products, these FVIII protein products could be ineffective if needed for breakthrough bleeding or surgery. This would likely be salient for novel FVIII variants with multiple modifications outside the FVIII B-domain, which are likely more immunogenic than modifications restricted to the B-domain; all current FVIII transgenes in Table 1 differ only in their B-domain replacement linker (69). If a patient is tolerized to a B-domain deleted FVIII transgene after gene therapy, the clinical decision making about using alternative FVIII protein products such as extended-half-life products would be similar to switching products after ITI with FVIII protein. Preclinical studies in inhibitor dogs using the canine orthologs of FVIII variants could evaluate this scenario for specific FVIII variants, as we did for the FIX-Padua (67, 133, 134).

### Non-factor Replacement for Hemostatic Prophylaxis and AAV Gene Therapy

Emicizumab and other non-factor therapies offer the potential for better and more convenient hemostatic prophylaxis for HA patients with inhibitors (3–6), including those undergoing ITI (135). Though the experience with emicizumab and FVIII protein ITI is currently limited (135), we would anticipate that a combination of a non-factor therapy hemostatic prophylaxis and AAV FVIII gene therapy ITI would be similarly attractive. This approach would have the advantage of limiting bleeds and thus exposure to non-transgene expressed FVIII or bypassing agents. Furthermore, the use of emicizumab would allow for both inhibitor titer and transgene FVIII levels to be closely monitored.

## Conclusion

Several AAV liver-directed gene therapy products for HA are moving through the clinical development pipeline (Table 1). To date, most clinical trials have mitigated the risk of inhibitor development by selecting only those subjects with heavy exposures to FVIII protein. Preclinical studies in a limited number of HA dogs (91, 94, 100) and other preclinical disease models (40, 42, 43) support the concept that AAV liver-directed gene therapy can harness the unique pro-tolerogenic properties of the liver, termed the liver tolerance effect (41), to induce peripheral immune tolerance to transgene FVIII. The potential advantages of our proposed AAV-mediated approach compared to standard ITI are summarized in Table 4. The ideal AAV FVIII vector for gene therapy ITI could be dosed low enough to avoid hepatotoxicities while still providing long-term hemostatic FVIII levels that, at a minimum, compare favorably to the hemostatic effect of emicizumab. Any planned immunosuppression must be evaluated in an immunocompetent animal model to determine if the specific regimen would negatively interfere with the immune tolerance induction to FVIII. This therapy would have dual benefits of eradicating the anti-FVIII antibodies while also providing continuous therapeutically relevant FVIII.

**TABLE 4:** Comparison of AAV-liver gene therapy versus protein-based FVIII for immune tolerance induction (ITI).

	**AAV-mediated**	**Protein-based**
Administration/frequency	IV/single injection	IV/3–7 days per week for years
Central vein catheterization	Not needed	Usually
Target population	Older children (>13 yo), likely similar to adult liver	Any age
Eligibility	No or low neutralizing antibodies titers to the vector capsid	All patients
Compliance	100%	<80%
Prophylaxis after inhibitor eradication	Endogenous expression of FVIII	FVIII replacement 2–3 times/week, indefinitely
Reversible in the event of allergic/anaphylaxis	No	Yes
Immunosuppression	May be needed to prevent/overcome cellular responses triggered by the vector capsid	Only in cases that failed multiple ITI attempts
Long-term complications	Potential insertional mutagenesis	Not applicable
Rates of success	No clinical data available. Preclinical studies in canine models showed high rates	60% in patients of good risk factors
Economic burden	High	High
Post ITI: maintenance of immune tolerance	None	FVIII protein 2–3 times/week

## Author Contributions

BS-J and VA conceived, drafted, and reviewed the manuscript.

## Conflict of Interest

The authors declare that the research was conducted in the absence of any commercial or financial relationships that could be construed as a potential conflict of interest.
